# Dietary Flavonoids as Cross-System Modulators of Hypertension and Intestinal Permeability

**DOI:** 10.3390/molecules31010048

**Published:** 2025-12-22

**Authors:** Jessica P. Danh, Andrew T. Gewirtz, Rafaela G. Feresin

**Affiliations:** 1 Department of Nutrition, Georgia State University, Atlanta, GA 30303, USA; jdanh1@gsu.edu; 2 Department of Chemistry, Georgia State University, Atlanta, GA 30303, USA; 3 Institute for Biomedical Sciences, Georgia State University, Atlanta, GA 30303, USA; agewirtz@gsu.edu

**Keywords:** polyphenols, tight junctions, inflammation, oxidative stress, renin–angiotensin system

## Abstract

Hypertension (HTN) and intestinal permeability (IP) are increasingly recognized as interrelated processes driven by shared oxidative and inflammatory mechanisms. This review synthesizes evidence linking HTN-induced vascular dysfunction to alterations in intestinal barrier integrity and explores the potential of dietary flavonoids as modulators of these pathologies. A narrative approach was used to synthesize findings from cellular, animal, and human studies that specifically address how flavonoids influence the molecular pathway connecting HTN and IP. Emerging evidence suggests that HTN-driven vascular injury, which is characterized by reduced nitric oxide bioavailability, increased reactive oxygen species, and pro-inflammatory signaling, contributes to tight junction disruption and increased IP. Mechanistic evidence indicates that flavonoids exert both direct antioxidant effects and indirect actions via the modulation of key cellular pathways. Preclinical and clinical data demonstrate that flavonoid-rich foods and isolated compounds can lower blood pressure, enhance endothelial function, and preserve intestinal barrier integrity by stabilizing tight junction proteins and attenuating pro-inflammatory signaling. Together, these findings highlight flavonoids as cross-system modulators that may mitigate HTN-associated increases in IP. Further research addressing sex, race, and age differences, as well as flavonoid bioavailability and dose optimization, is needed to clarify their translational potential.

## 1. Introduction

High blood pressure (BP), or hypertension (HTN), is a major modifiable risk factor for cardiovascular disease (CVD) [1], the leading cause of mortality worldwide [2]. In the United States alone, CVD was responsible for 919,032 lives lost in 2023, representing nearly one in every three deaths [3]. In a meta-analysis of 61 prospective studies, each 20 mmHg increase in systolic BP (SBP) and 10 mmHg increase in diastolic BP (DBP) was associated with a two-fold increase in mortality from stroke, ischemic heart disease, and other vascular-related causes [4]. These findings underscore the need to better understand the pathological mechanisms underlying HTN and to develop strategies that mitigate its onset and progression. While several pharmacological therapies effectively reduce BP and CVD risk, treatment typically begins only after HTN is established (SBP ≥ 140 mmHg or DBP ≥ 90 mmHg, unless other disease is present) [5]. Moreover, adherence to antihypertensive medication can be limited by costs, adverse effects, or limited access to care [6,7,8]. Thus, a greater emphasis on early prevention through modifiable lifestyle and dietary interventions is warranted.

Diet is a well-established determinant of cardiovascular health. Indeed, a recent meta-analysis comparing 13 dietary approaches found that the DASH (Dietary Approach to Stop Hypertension) diet was most effective for lowering SBP and DBP [9]. The DASH diet emphasizes fruits, vegetables, and low-fat foods while reducing saturated fat, total fat, and cholesterol [10]. Fruits and vegetables contain phytochemicals such as polyphenols which contribute to cardiovascular protection [11]. Among these, flavonoids, a major subclass of polyphenols, have demonstrated BP-lowering effects, which may be due to enhanced antioxidant activity, and increased production and bioavailability of nitric oxide (NO), a potent vasodilator [12,13,14].

Emerging evidence suggests that gut health, including both the gut microbiome and the integrity of the intestinal barrier, plays an important role in BP regulation [15,16,17,18]. Several pathways have been proposed to link the intestine to HTN, including microbial metabolite production (e.g., short-chain fatty acids), shifts in gut microbial composition such as alterations in *Akkermansia municiphilia*, and changes in immune and neurohumoral signaling [19,20,21,22]. While these mechanisms collectively highlight the multifaceted nature of gut-vascular interactions, growing attention has centered on the intestinal barrier as a critical interface influenced by hypertensive physiology.

Recent studies report that HTN is associated with increased intestinal permeability (IP), allowing excessive translocation of luminal antigens, bacteria, and inflammatory molecules into systemic circulation. [23,24]. Whether HTN induces IP or vice versa remains uncertain; however, increased IP, often termed “leaky gut,” has been linked to systemic inflammation and the development of metabolic disorders, both of which can exacerbate hypertensive pathology [25].

Notably, the discovery that the renin–angiotensin system (RAS) is not limited to systemic circulation, but also operates locally within the intestine provides a unifying mechanism linking vascular dysfunction to barrier disruption [26]. Angiotensin (Ang) II can directly influence both vascular tone and epithelial tight junction (TJ) regulation, positioning RAS at the intersection of HTN and IP [27]. This dual involvement offers a biologically coherent rationale for focusing on RAS-driven pathways in understanding how these conditions reinforce one another.

Therefore, the purpose of this review is to highlight the association between HTN and IP and to examine the role of dietary flavonoids as modulators of this RAS-mediated relationship. We begin with an overview of the intestinal barrier, with emphasis on TJs as key determinants of barrier integrity. Next, we consider how the RAS, the principal regulator of BP and fluid balance, contributes to IP alterations. Finally, we explore the potential of flavonoids as therapeutic bioactives in the prevention and management of HTN-associated barrier dysfunction.

## 2. Methods

A targeted literature search was conducted in PubMed through October 2025 and restricted to English-language publications. Search terms included combinations of keywords related to IP (“tight junction,” “claudin,” “zonula occludens,” “occludin,” “intestinal permeability”), HTN (“hypertension,” “angiotensin II,” “renin–angiotensin system”), and dietary bioactives (“flavonoids,” “polyphenols”), along with mechanistic modifiers (“oxidative stress,” “inflammation”). Boolean operators (e.g., AND, OR) were used to capture intersections relevant to this review, such as “hypertension AND intestinal permeability,” “angiotensin II AND intestinal barrier,” and “flavonoids AND tight junction.”

Studies using human participants, animal models, or cell culture systems were included when they examined (1) the effects of HTN or Ang II on IP, TJ regulation, or intestinal barrier function, or (2) the mechanistic or physiological actions of flavonoids relevant to epithelial or vascular redox and inflammatory pathways. Because the objective of this review was to integrate mechanistic evidence across biological levels rather than to conduct a systematic evaluation of all available studies, no formal risk-of-bias assessment or structured data extraction was performed. This approach is consistent with narrative and mechanistic reviews aimed at synthesizing conceptual frameworks and identifying emerging research directions.

## 3. The Intestinal Barrier: Gateway to the Host

When discussing the intestinal barrier, it is important to note that it is the result of a healthy intestinal mucosa which comprises multiple elements working in tandem to allow for the uptake of nutrients while preventing the translocation of toxic luminal substances into systemic circulation. Averaging approximately 26 feet in length in the human intestine, it serves as the longest interface between the external and internal environments of the host [28].

The intestinal mucosa is made up of four distinct histological layers. From the outermost to the innermost, there are four layers: the serosa, muscularis propria, the submucosa, and the mucosa. The mucosa itself contains three sublayers: the muscularis mucosae is the deepest layer and is composed of smooth muscles to assist with glandular excretions; the lamina propria is superficial to the muscularis mucosae and is composed of connective tissues housing a network of vessels, nerves, and lymphatic tissue which supports nutrient transportation and immune system function; and finally, the epithelial layer is the most superficial layer and lines the entirety of the intestinal mucosa, making itself directly exposed to the lumen. Additionally, a mucus layer within the lumen lines the epithelial layer to protect from intestinal pathogenic bacteria while nourishing symbiotic bacteria, thereby maintaining homeostasis of the intestinal flora [29].

Our main area of interest in this review is found in the intestinal epithelial layer. Progenitor cells in the crypts of the intestinal villi give rise to five cell types that make up this continuous monolayer of intestinal epithelial cells (IECs): the absorbative enterocytes, the mucus-producing goblet cells, the hormone-producing enteroendocrine cells, the antimicrobial-producing Paneth cells, and the antigen-sampling microfold cells. These IECs are tightly bound to adjacent cells by intercellular junctional complexes which regulate paracellular transit of luminal substances. They include desmosomes, adherens junctions, and TJs. While cell-to-cell adhesion is just one of the functions that desmosomes and adherens junctions perform, their roles have been reviewed [30,31] and will not be discussed here.

**Figure 1:**
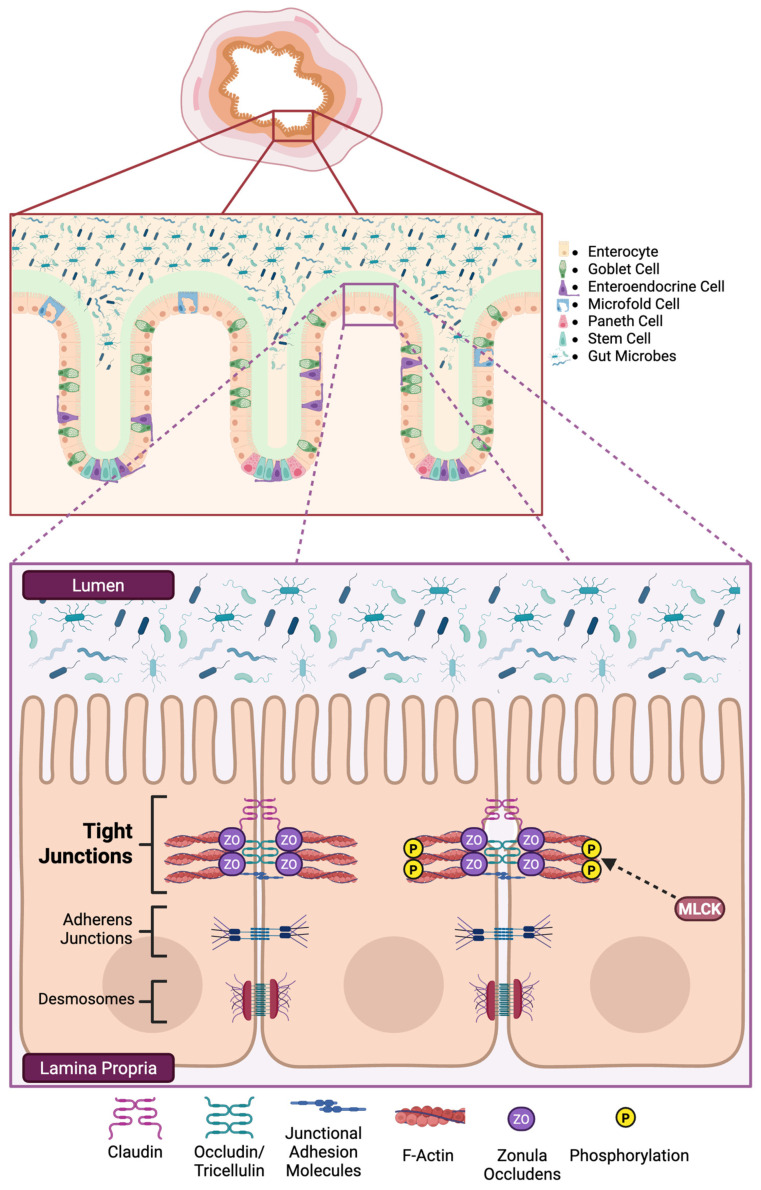
The structure of the intestinal mucosa and epithelial junctional complexes. Tight junctions, composed primarily of occludin, claudins, junctional adhesion molecules, and zonula occludens (ZO) proteins, form the apical seal that regulates paracellular permeability. Beneath them, adherens junctions and desmosomes provide lateral adhesion and structural stability. Phosphorylation of ZO proteins by myosin light-chain kinase (MLCK) promotes cytoskeletal contraction and junctional opening, leading to increased paracellular flux. Created with BioRender.com.

Tight junctions are found near the apical surface of IECs and perform a crucial role in the regulation of intestinal paracellular permeability by selectively controlling the flow of water, ions, and other molecules. These complexes are formed by several integral and scaffolding proteins: claudins (CLDN), occludin (OCLN), junctional adhesion molecules (JAMs), tricellulin, and zonula occludens (ZO). We will briefly review each protein and the important roles each perform in forming the TJ complex. The structure of the intestinal mucosa and TJ proteins can be found in Figure 1.

### 3.1. Claudins

The CLDN family of proteins consists of 26 and 27 members in humans and rodents, respectively [32]; however, only a subset of these proteins is expressed in the intestine. For example, human colons express CLDN-1, -2, -3, -4, -7, -8, -12, and -15 [33], while CLDN-18 is uniquely expressed in alveolar epithelial cells [34]. Claudins are 18–27 kDa tetraspan membrane proteins that consist of two extracellular loops, a short cytosolic loop, and amino- and carboxy-terminal ends that project into the cytoplasm [35]. This is important to note because the first extracellular loop is the main determinant in creating the charge and size selective pore [36], while the second extracellular loop is responsible for maintaining the CLDN-CLDN interaction with adjacent cells. Interestingly, each CLDN isoform has been classified as either tight, leaky, or pore-forming. Moreover, the pair of CLDNs formed when adjacent cells form TJs can further be ascribed as tight or leaky depending on the pairings [35]. The carboxy-terminal ends of CLDN proteins contain the PDZ-binding motif, YV, which enables them to bind to the PDZ domains of the scaffolding protein, ZO—crucial for its incorporation into the TJ [36,37,38].

Claudins are critical to maintaining barrier function as their absence or disruption in TJs have been implicated in disease [39]. For example, interferon (IFN)-γ-induced internalization of CLDN-1 and CLDN-4, classified as tight or sealing proteins, is suggested to contribute to the barrier dysfunction seen in intestinal barrier disease (IBD) [40]; whereas CLDN-2, a pore-forming protein, was upregulated in IBD [41]. In contrast, TJs may potentially function properly without OCLN. For example, morphological changes to TJ complexes and decreases to intestinal barrier function were not observed in OCLN^−/−^ mice [42]; however, this may be due to compensatory mechanisms increasing tricellulin production, taking up the absence of OCLN [43].

### 3.2. Occludin

Occludin was first identified by Furuse et al. in 1993 [44]. It is a 65 kDa tetraspan membrane protein containing a short cytosolic loop separating two extracellular loops rich in tyrosine and glycine residues which interact with the extracellular loops of OCLN of adjacent IECs. The long carboxy-terminal tail of OCLN projects into the cytosol to interact with scaffolding proteins such as ZO, and is rich in serine, threonine, and tyrosine residues, making it the target of protein kinases [37,44]. In fact, a study examining the properties of OCLN in Madine–Darby Canine Kidney (MDCK) cells found that differential phosphorylation of OCLN may be a mechanism to alter its function and localization [45]. For example, the proper assembly of TJs requires that OCLN be phosphorylated on its serine residues [46]; however, phosphorylation of the tyrosine residues was shown to disrupt the assembly. Indeed, hydrogen peroxide-induced phosphorylation of tyrosine residues results in a disturbance in the interaction between OCLN and ZO-1 in Caco-2 cells [47].

### 3.3. Tricellulin and Junctional Adhesion Molecules

Tricellulin is another tetraspan transmembrane protein with a carboxy-terminal that is 32% identical to OCLN [36]. It is found ubiquitously in epithelial cells and most notably at tricellular tight junctions, where three cells meet. Junctional adhesion molecules are 43 kDa glycosylated transmembrane proteins that function as cell adhesion receptors and display two V-type extracellular domains typical of immunoglobulin (Ig) G loops for which they are apart [48,49]. Also included is a single transmembrane region and a short carboxy-terminal which projects into the cytosol [37]. The JAMs associated with intestinal barrier function include JAM-A and JAM-4 [50,51,52]. Interestingly, JAM-A appears to have an important role in intestinal barrier function as seen in patients with Crohn’s Disease (CD) and ulcerative colitis (UC) [53]. Additionally, JAM-A^−/−^ mice demonstrated increased IP to dextran 4 h-post oral gavage compared to control animals [54].

### 3.4. Zonula Occludens

Zonula occludens are 130-225 kDa proteins that function as scaffolding proteins and are critical for the formation of TJs. There are three main isoforms: ZO-1, ZO-2, and ZO-3. The PDZ1 domain of ZO-1 binds to CLDNs while its PDZ2 domain can bind to both ZO-2 and ZO-3, suggesting that the ZOs form their own complex [55,56]. Although OCLN has been observed to anchor to the actin cytoskeleton directly, ZO-1 generally serves as a link between the two [57]. Additionally, it can interact with the PDZ domain binding motif of JAM-A [58]. Similarly, ZO-2 can bind both CLDN and OCLN on its amino-terminal end and to actin on its carboxy-terminal end [56]. The ZO proteins act as adaptors to enable the assembly of TJ complexes which interact with the peri-junctional actomyosin ring. This allows for the circumferential contractions that regulate paracellular permeability by allowing or barring the passage of luminal contents. These contractions are regulated by the phosphorylation of myosin light chain (MLC) by myosin light chain kinase (MLCK) [59,60,61].

Altogether, CLDNs, OCLN, JAMs, tricellulin, and ZO form the essential TJ complex that serves as gatekeepers of paracellular transport and for which the overall integrity is paramount in maintaining intestinal barrier function to prevent increases on intestinal permeability.

## 4. RAS: A Link Between Hypertension and Intestinal Permeability

The RAS is well-known for its central role in regulating BP and fluid balance through its main effector peptide, Ang II. Its relevance to this review extends beyond hemodynamic control: Ang II signaling, particularly through the Ang II type 1 receptor (AT_1_R), drives oxidative stress, inflammation, and cytoskeletal contraction, all of which contribute to TJ disruption and increased IP. Because these Ang II-mediated mechanisms mirror those that underlie hypertensive injury, the RAS represents a unifying pathway linking HTN to intestinal barrier dysfunction. To contextualize these interactions, we first outline the physiological actions of the RAS before discussing its implications for IP.

The formation of Ang II first begins with angiotensinogen which is produced by the liver. Circulating renin from the kidneys will hydrolyze angiotensinogen to form Ang I. Angiotensin converting enzyme (ACE), found predominantly in the endothelial cells of pulmonary circulation, further hydrolyzes Ang I to form Ang II. Ang II will then circulate and bind to its main receptor, AT_1_R, a G protein-couple receptor (GPCR), on vascular smooth muscle cells to exert its vasoactive effects leading to the downstream phosphorylation and activation of MLC. This triggers cross-bridge cycling, thereby inducing vasoconstriction of the vessel. Further promotion of MLC phosphorylation by Ang II is through its activation of Rho-Kinase which inhibits MLC phosphatase. Additionally, Ang II stimulates the release of aldosterone from the adrenal cortex which increases sodium retention in the kidneys and promotes thirst by the hypothalamus, thereby increasing blood volume. Altogether, these effects lead to the physiological rise in BP.

Ang II can also bind to angiotensin type 2 receptor (AT_2_R), which serves as a counter-regulatory pathway to AT_1_R signaling [62]. Unlike AT_1_R, AT_2_R activation stimulates NO and cyclic guanosine monophosphate (cGMP) production to mediate vasodilation, and promotes anti-proliferative, anti-fibrotic, and anti-inflammatory actions [63,64,65]. Together, these opposing pathways highlight the balance between pressor (AT_1_R) and depressor (AT_2_R) mechanisms within the RAS.

Under pathological conditions that lead to HTN, hyperactivity of RAS increases Ang II production which has been shown to promote oxidative stress and inflammation alongside increases in BP. Major sources of Ang II-induced reactive oxygen species (ROS) are produced by nicotinamide adenine dinucleotide phosphate (NADPH) oxidases (NOX), xanthine oxidase (XO), and the mitochondria. This increase in superoxide anion formation not only damages cellular structures but depletes circulating NO which inhibits the vascular endothelium-dependent vasodilatory response. Ang II activation of AT_1_R activates the IκB kinase (IKK) complex which leads to the downstream activation and translocation of nuclear factor kappa-light-chain-enhancer of activated B cells (NFκB), a key redox transcription factor involved in the vascular inflammatory process responsible for regulating gene expression of pro-inflammatory cytokines such as tumor necrosis factor (TNF)- α, interleukin (IL)-6, IL-1β as well as the pro-inflammatory enzyme cyclooxygenase (COX)-2. NFκB can also increase AT_1_R expression, thereby promoting a positive feedback mechanism of inflammation and oxidative stress.

Altogether, chronic AT_1_R activation promotes vascular dysfunction by sustaining vasoconstriction, reducing NO bioavailability, and exposing the endothelium to persistent oxidative and inflammatory stress. Excessive ROS and pro-inflammatory cytokines impair endothelial integrity, blunt endothelium-dependent vasodilation, and accelerate vascular remodeling—hallmarks of hypertensive vascular injury. Importantly, many of these same Ang II-mediated mechanisms that disrupt vascular function also influence epithelial TJ stability and intestinal barrier regulation, providing mechanistic rationale for examining Ang II-induced changes in IP. Extensive reviews have examined the role of the RAS in HTN, particularly its contributions to oxidative stress and inflammation; we direct readers to reviews by Arendshorst et al. [66] and Tanase et al. [67] for comprehensive discussions on these mechanisms.

### 4.1. Angiotensin II Alters Intestinal Permeability and Tight Junction Proteins In Vivo

The discovery of local RAS makes it clear that Ang II can also bind to IECs through the classical (AT_1_R) and alternative (AT_2_R) RAS pathways [26]. Despite this, few studies using the Ang II-infusion model of HTN, which makes up nearly 50% of National Institute of Health-sponsored HTN research, focus on its association with IP [68]; however, they each converge on similar conclusions: Ang II-induced HTN was associated with detrimental alterations to the intestinal barrier and increase IP. To specifically isolate the contributions of Ang II to IP, Table 1 summarizes animal studies using Ang II-infusion models in which IP or barrier-related outcomes were directly assessed. Only a limited number of studies have examined these endpoints within this model, underscoring a notable gap in the literature; by contrast, more extensive work has been performed in SHRs, where HTN arises through multiple mechanisms beyond Ang II alone.

Santisteban et al. [23] employed two rat models of HTN to investigate the association between IP and HTN. In the first model, IP and TJ protein expression from 20-week-old and four-week-old spontaneously hypertensive rats (SHRs) were compared to normotensive age-matched Wistar–Kyoto rats (WKY). The group found that older SHRs with established HTN had significantly increased IP as measured by plasma FITC (fluorescein isothiocyanate)-dextran assay compared to WKYs but no difference between the younger cohort was observed. Furthermore, protein expressions of OCLN, ZO-1, and CLDN-4 were significantly decreased in the small intestine of adult SHR compared to WKY, with comparable decreases in the proximal colon. Interestingly, they found that the TJ proteins were also significantly lower in the young SHRs compared to young WKYs in both the small intestine and the proximal colon suggesting that alterations in TJ protein expression may precede changes in IP before the establishment of HTN. To verify that IP is altered in other models of HTN, the group infused Ang II (200 ng/kg BW/min) in Sprague-Dawley (SD) rats and found that permeability significantly increased alongside a decrease in the same TJ proteins. The same group later investigated potential neural mechanisms involved in HTN using the same Ang II infusion model, and while permeability was not measured, they reported pathological changes in the gut wall with Ang II infusion whereby muscularis thickness and fibrotic area increase while goblet cells decreased [69].

Similar findings were reported by Kim et al. [24] after infusing C57BL/6 mice with Ang II (1000 ng/kg BW/min) for four weeks. This group found that mice with established HTN had significantly increased IP as measured by serum FITC-dextran assay compared to control, and that further analysis of TJ gene expression demonstrated a significant decreased in OCLN and ZO-1. While CLDN-4 was also decreased, it was not significant compared to control. This agrees with a later study by Kaye et al. [70] that used a slow-pressor dose of Ang II (173 ng/kg/min) alongside the absence of resistant starches in C57BL/6J mice to show that a mild hypertensive stimulus alongside a fiber-poor diet can alter the intestinal barrier by decreasing gene expression of tight junction protein (TJP)-1 (also known as ZO-1).

A more recent study by Majumder et al. [71] demonstrated the role of toll-like receptor (TLR)4 in HTN-induced gut hyperpermeability by using the C3H/HeJ^Lps-d^ strain [nonfunctional TLR4; referred to as TLR4 mutant (TLR4M)] and the C3H/HeOuJ strain [functioning TLR4; referred to as TLR4 normal (TLR4N)]. Animals were infused with Ang II (1000 ng/kg/min) for four weeks and alterations to TJ proteins via immunolocalization, IP via FITC-dextran, and gut bacterial translocation were measured. The group found that OCLN, ZO-1, and CLDN-1 immunolocalization decreased in the colon of TLR4N mice yet remained the same in TLR4M. This was accompanied by increases in both IP and bacterial translocation in the TLR4N group compared to TLR4M, suggesting an important role for TLR4 in Ang II-induced gut dysfunction.

Although many studies examining the effects of Ang II or RAS-related mechanisms on IP and TJ proteins in vivo arise from IBD models, they provide valuable insight into how RAS hyperactivation influences intestinal barrier function outside of classical hypertensive contexts. He et al. [27] reported that hyperactivation of RAS promotes colonic inflammation mediated through Ang II activation of the Janus kinase/signal transducer of activated transcription (JAK/STAT) pathway in RenTgMK mice, a transgenic model in which renin is overproduced. As the rate-limiting step in RAS, an over production in renin would invariably lead to an increase in Ang II and results in heightened AT_1_R activation. In addition to driving vasoconstriction, AT_1_R activation increases cytokine production; when cytokines such as pro-inflammatory IL-6 bind their cell surface receptors, they trigger JAK-mediated phosphorylation of STAT proteins, including downstream pro-inflammatory gene transcription. Consistent with this, RenTgMK mice displayed heightened colonic inflammation and increased MLCK expression, suggesting disruption of normal intestinal barrier function.

More recently, Pan et al. [72] demonstrated that high-fat diet-induced obesity also activates local RAS signaling in the colon of C57BL/6J mice. Mice fed a high-fat diet (60% kcal from fat) for 10 weeks exhibited significant increases in colonic expression of AT_1_R, angiotensinogen, and renin compared with low-fat diet controls; these effects were amplified following 2,4,6-trinitrobenzensulphinic acid (TNBS)-induced colitis. This upregulation of RAS components was accompanied by increased IP, measured by FITC-dextran flux, and reduced OCLN protein expression. Importantly, treatment Losartan, a AT_1_R antagonist, attenuated these changes by reducing AT_1_R expression, increasing AT_2_R expression, and partially restoring barrier integrity and OCLN levels. These findings further support a role for local RAS activation in driving intestinal barrier dysfunction.

### 4.2. Increased Intestinal Permeability in Human Hypertension

A “gold standard” approach to determining IP in humans has yet to be established. While methods measuring the urinary excretion of tracer molecules such as lactulose and mannitol are considered accurate [73], these methods are often time-consuming and lack standardization. Measurement of endogenous proteins and translocation of gut-derived contents have also been utilized as biomarkers for IP. We will discuss several commonly employed measures. Intestinal fatty acid binding protein (I-FABP) facilitates the absorption and transport of fatty acids and is expressed in the epithelial cells of the intestinal mucosae of the small and large intestine. Following intestinal injury such as in cases of celiac disease, there is an associated increase in plasma I-FABP and has therefore been used as a non-invasive marker of IP and inflammation [74]. Lipopolysaccharide (LPS) is an endotoxin found on the cell envelope of Gram-negative bacteria and can translocate from the intestine with increased IP. Increases in LPS will induce the production of LPS binding protein (LBP), an acute-phase protein, which binds to LPS to trigger an immune response [75]. Both LPS and LBP are used as measures of IP. Similarly, D-lactate is largely produced by gut bacteria and its elevation in the blood has been associated with increased IP and intestinal injury [76]. Zonulin has been described as a reversible regulator of IP by modulating TJ proteins and can be measured in the blood or fecal samples [77]. When elevated, zonulin promotes the disassembly of TJs and therefore increases IP. Diamine oxidase (DAO) functions to oxidize histamine and is widely distributed in tissues with high presence on the apical surface of mature enterocytes. Intestinal injury has been associated with increased serum DAO levels and is therefore considered a marker of IP [78].

Using the markers discussed above, several studies have observed an association between increased markers of IP with HTN in humans. For example, in a study of 357 patients with (*n* = 251) and without (*n* = 106) HTN, serum DAO, serum LPS, and serum D-lactate were measured to determine intestinal barrier function [79]. They observed significant increases in serum DAO and LPS in patients with HTN compared to those without, but not serum D-lactate. This increase in serum LPS agrees with previous findings by Kim et al. [24] who reported similar results in the plasma of a smaller cohort (*n* = 40). Only a small number of human studies have directly evaluated IP in the context of primary HTN. Table 2 summarizes the three available cohorts that have assessed circulating markers of gut barrier dysfunction (zonulin, LPS, DAO, D-lactate, I-FABP) in individuals with elevated BP. Although limited, these studies consistently report higher concentrations of at least one permeability marker in hypertensive or high-BP groups, supporting a potential clinical association between HTN and impaired intestinal barrier function.

While these biomarkers provide accessible, non-invasive methods for estimating IP, their interpretation requires caution as each has demonstrated limitations. For example, circulating LPS is widely used as a surrogate marker for increased IP but it has been criticized for its methodological constraints, most notably poor assay precision, susceptibility to contamination, and lack of standardization across laboratories and kits [81]. LBP has been proposed as a more stable alternative to LPS; however, as an acute-phase reactant, its levels may increase in response to systemic inflammation independent of barrier dysfunction [82]. Additionally, commercially available ELISA kits for serum zonulin have been criticized for lacking specificity as they often detect related proteins rather than true zonulin, or pre-haptoglobin-2 [83]. I-FABP, while useful in conditions of severe illness, shows reduced sensitivity in healthy or mildly diseased populations, limiting its utility as a generalized permeability marker [73,84]. Collectively, these limitations highlight the need to interpret biomarkers within appropriate clinical and physiological contexts and caution against relying on any single measure to assess IP.

### 4.3. Inflammation and Oxidative Stress: Drivers of Ang II-Induced Permeability

Markers of inflammation and oxidative stress are well-known for their deleterious effects on the intestinal barrier and increasing IP. Indeed, as byproducts of Ang II activation of AT_1_R, inflammation and oxidative stress may be one of the primary pathways by which intestinal barrier function is altered in cases of HTN. For example, a recent study by Takashina et al. [85] demonstrated the role of Ang II in regulating the paracellular transport of Cl^−^ by increasing CLDN-7 protein and gene expression in MCE301 mouse colonic cells. Cells were treated with 10 μM of Ang II alone or in addition to Losartan, an AT_1_R blocker, or PD123319, an AT_2_R blocker. Losartan inhibited Ang II-induced increased in CLDN-7, but not PD123319, suggesting that the increase in CLDN-7 is mediated through AT_1_R activation. Gene expression of other TJ proteins was also measured (CLDN-1, CLDN-2, CLDN-4, OCLN, ZO-1) but was not significantly altered. Further measurements revealed an increase in CLDN-7 co-localization with ZO-1 in Ang II-treated cells alongside a significant decrease in transepithelial electrical resistance (TEER), a measurement of paracellular permeability in which a decrease indicates greater permeability. Paracellular permeability as measured by lucifer yellow was not significantly different among groups, suggesting Ang II alters permeability to ions rather than small solutes. Further investigation showed an increase in nuclear fractions of the phosphorylated-p65 (p–p65) subunit of NF-κB as well as an increase in the promoter activity of CLDN-7; however, both were inhibited when treated with BAY 11-7082, an NF-κB inhibitor. Altogether, their findings suggest Ang II-induced increases in CLDN-7 expression are mediated through the NF-κB pathway. This is important to note as the NF-κB pathway is a central mediator of the inflammatory response.

He et al. [27] also investigated the mechanisms by which Ang II promotes inflammation by treating HCT116 cells, a human colonic epithelial cell line, with 100 nM of Ang II. Within 15 min, they observed activation of JAK2, STAT1, and STAT3. After 16 h, there was an increase in transforming growth factor (TGF)- β1, MLCK, and p53 upregulated modulator of apoptosis (PUMA). This was accompanied by an increase in gene expression of pro-inflammatory markers such as TNF-α, IL-6, and IL-1β. Production of these markers were attenuated with tofacitinib, a JAK inhibitor, suggesting that colonic inflammation was promoted through an Ang II-JAK2/STAT pathway.

Other studies have also demonstrated the direct effect of inflammatory cytokines and superoxide on IP and TJ proteins. For example, while low, physiological doses of TNF-α do not appear to alter intestinal barrier function, Wang et al. [86] found that Caco-2 cells, a human cell line commonly used to mimic the intestinal epithelium, sequentially treated with 10 ng/mL IFN-γ then 2.5 ng/mL TNF-α rapidly reduced TEER and increased 3 kDa dextran flux indicating an increase in permeability. Interestingly, they found that the insult did not degrade ZO-1, OCLN, or CLDN-1; rather, the proteins were reorganized such that TJ proteins at the cell-to-cell interface became irregular and the intracellular pools of OCLN and CLDN-1 were expanded. Insult with IL-1β has also been shown to decrease TEER in a dose-dependent manner [87]. As seen before, this effect appears to be mediated through the activation of NF-κB as treatment with pyrrolidine dithiocarbamate (PDTC), an NF-κB inhibitor, prevented IL-1β induced decreases in TEER and OCLN expression. Xanthine oxidase is responsible for the catabolism of purines; more specifically, it catalyzes the breakdown of hypoxanthine and xanthine into uric acid using oxygen as a substrate. Reducing oxygen in this process results in the formation of superoxide anions which, when produced in excess, leads to a buildup in ROS and thus, oxidative stress. When Caco-2 cells were treated with XO and xanthine to induce oxidative stress, Rao et al. [47] observed a decrease in TEER and an increase in mannitol flux, indicating increased permeability. This appears to have been mediated through oxidative stress-induced phosphorylation of the tyrosine residues of OCLN.

### 4.4. Hypertensive Medications Improve Intestinal Barrier Function

The studies discussed thus far indicate a strong association between increased IP and HTN. Consistent with this, several antihypertensive drugs that target components of the RAS also appear to preserve intestinal barrier function, suggesting that Ang II-mediated signaling contributes directly to epithelial injury. For example, SHR treated with Captopril, an ACE inhibitor, for four weeks exhibited significantly reduced mean arterial pressure and lower IP, along with improved intestinal morphology, including decreased muscle thickness and increased villus height [23]. A complementary study by Li et al. [88] reported that eight weeks of Captopril treatment in SHR increased protein expression of TJP1 and OCLN as well as improved mucosal structure, even though permeability was not directly assessed. Similar protective effects have been observed with Enalaprilat, which preserved ZO-1 localization and prevented OCLN internalization while reducing FITC-dextran flux in dextran sodium sulfate (DSS)-induced colitis mice [89].

Across Ang II receptor blockers (ARBs), comparable patterns emerge. Losartan has been shown to reduce IP in TNBS-induced colitis mice, enhance OCLN and ZO-1 gene expression lowering circulating LPS in SHR, and decrease colonic permeability in LPS-treated Sprague-Dawley rats [90,91,92]. Collectively, these findings demonstrate that both ACE inhibitors and ARBs consistently attenuate epithelial barrier dysfunction across diverse injury models, strengthening the mechanistic link between Ang II-AT_1_R signaling and IP. For additional context, Lucas et al. [93] provide an in-depth review of antihypertensive e medications and their effects on intestinal barrier integrity. Table 3 summarizes the studies reporting the effects of RAS-targeted medications on IP and related outcomes.

## 5. Dietary Flavonoids: Modulators of Hypertension and Intestinal Permeability

While pharmacological therapies remain the cornerstone in the management of HTN, lifestyle changes such as dietary improvements offer a safe and cost-effective strategy to prevent or delay disease progression. Accumulating evidence supports the role of plant-based diets in the reduction in SBP and DBP [95,96]. Plant foods contain a plethora of bioactive components with demonstrated health benefits. Among them are polyphenols, secondary metabolites that play a role in plant defense against environmental factors such as ultraviolet light, water availability, pests, or physical injuries [97]. Although polyphenols are not essential for growth and survival, numerous studies have demonstrated clear beneficial effects in reducing all-cause mortality and major chronic diseases [98,99,100]. These hydroxyl group-carrying compounds are broadly categorized into four classes, flavonoids, phenolic acids, stilbenes, and lignans, based on the number of phenol rings present and the structural elements that bind them.

Flavonoids have long been a focus in dietary bioactives research due to their widespread presence in plant-based foods and their diverse biological activities. Of the over 10,000 flavonoids identified, several hundred occur in the edible parts of plants found in the human diet [101]. These flavonoid-rich foods range from fruits and vegetables, nuts and seeds, and herbs and legumes. Given their derivation from plants, teas, wine, and beer are also sources of flavonoids.

### 5.1. The Structure and Metabolism of Flavonoids

**Figure 2:**
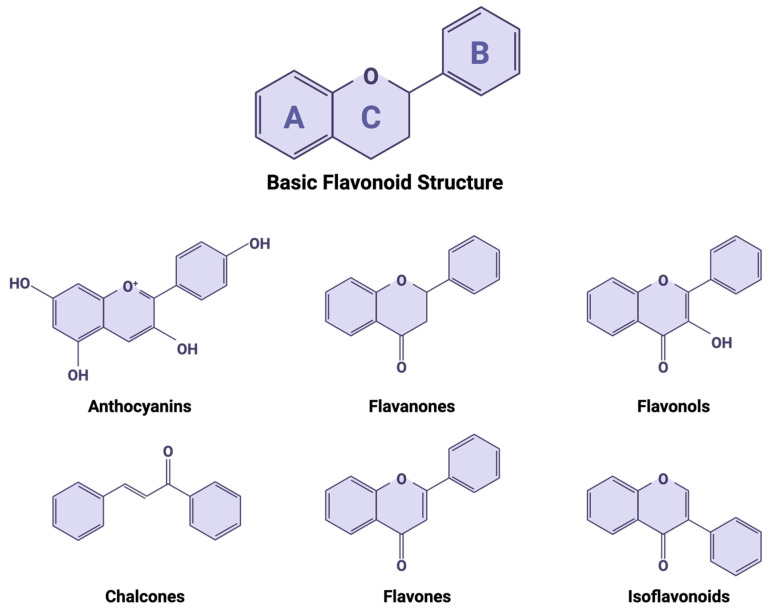
The basic structure of a flavonoid and flavonoid classes. Created with BioRender.com.

Recent reviews have thoroughly discussed the structure and characteristics of flavonoids [102,103]; therefore, we will only provide a brief discussion here. The variation in the structure of flavonoids gives rise to their differences in bioavailability, metabolism, and bioactivity. Flavonoids share a common 15-carbon (C6-C3-C6) backbone, consisting of two benzene rings, A and B, joined by a heterocyclic pyran ring, C, containing an oxygen atom (Figure 2) [102]. The degree of saturation of the heterocyclic pyrene ring further categorizes flavonoids into one of six major subclassifications: flavones, flavonols, flavan-3-ols, isoflavones, flavanones, and anthocyanidins.

Flavonoids are naturally found in foods in their glycosylated forms as it improves solubility, distribution, and metabolism [103]. During digestion, flavonoid glycosides must be deglycosylated before absorption can occur in the small intestine, a process highly dependent on the position of the sugar substitution and therefore only accounts for a small portion of flavonoid absorption [104,105]. Flavonoid glycosides that reach the colon undergo microbiota-mediated hydrolysis and fermentation prior to absorption or are otherwise voided in the feces [104]. Once absorbed, flavonoids enter portal circulation and reach the liver where they undergo phase I and phase II metabolism resulting in more polar metabolites [106]. These metabolites are either excreted in the urine via the kidneys or reach their target tissue to exert an array of biological effects. It should be noted that flavonoids are seldom found in the bloodstream in their parent form; rather, their metabolites are responsible for much of the activity attributed to flavonoids [100].

### 5.2. The Bioactivities of Flavonoids

While traditionally recognized for their antioxidant properties, flavonoids are now understood to influence health through multiple, distinct biological functions. Importantly, however, the biological effects attributed to flavonoids must be interpreted within the context of their metabolism and realistic human exposure. Typical dietary intake of total flavonoids in the United States ranges from approximately 300–500 mg/1000 kcal/d and the compounds that circulate human plasma are predominantly phase II conjugated metabolites rather than the parent compounds [107,108,109]. These metabolites, shaped by both gut microbial and hepatic metabolism, differ in bioavailability and activity from the compounds often used in experimental systems. Therefore, discussions on flavonoids bioactivity must consider not only their mechanistic potential but also the exposure levels.

#### 5.2.1. Antioxidant Activity

Free radicals and ROS, such as superoxide (O_2_•^−^) and hydrogen peroxide (H_2_O_2_), are natural byproducts of aerobic respiration and energy production. To limit oxidative damage, the body relies on endogenous antioxidant mechanisms, including superoxide dismutases (SOD), glutathione peroxidases (GPx), and catalase (CAT). However, under conditions of stress, injury, or disease, excessive ROS production can overwhelm these defenses, leading to increased susceptibility to cellular damage.

**Figure 3:**
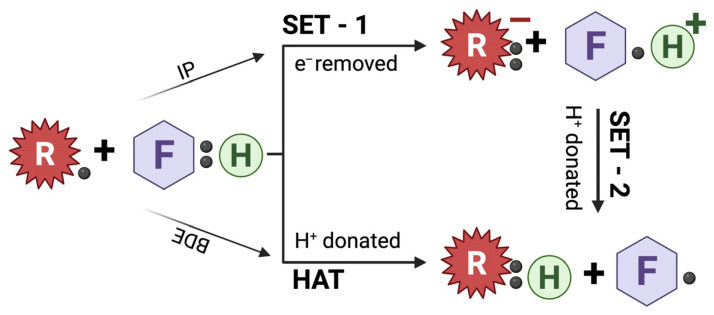
Antioxidant mechanisms of flavonoids via single-electron transfer (SET) and hydrogen-atom transfer (HAT) pathways. Schematic representation of the major redox mechanisms by which flavonoids neutralize free radicals. In the SET mechanism, an electron (e^−^) is transferred from the flavonoid (F:H) to the radical (R), forming a protonated radical intermediate (F−H^+^) that subsequently donates a proton. In the HAT mechanism, the flavonoid directly donates a hydrogen atom (H) to neutralize the radical. The predominant pathway depends on the flavonoid’s ionization potential (IP) and bond dissociation enthalpy (BDE). Created with BioRender.com.

Flavonoids exert potent antioxidant activity primarily through single electron transfer (SET) or hydrogen atom transfer (HAT) mechanisms. The predominant pathway depends on ionization potential, the energy required to remove an electron, and bond dissociation enthalpy, the energy required to break an O—H bond. In the SET pathway, a flavonoid donates its electron to a free radical, whereas in the HAT pathway, it donates a hydrogen atom and its electron. Both processes stabilize ROS and generate less reactive radical products. A schematic representation of the SET and HAT pathways can be seen in Figure 3. The antioxidant potency of flavonoids is strongly influenced by their chemical structure, particularly the number and position of hydroxyl groups, with substitutions on the B ring conferring greater radical scavenging capacity [13].

Transition metals such as iron (Fe) and copper (Cu) are essential micronutrients required in trace amounts for critical biological functions. In humans, over 60% of iron is found in hemoglobin for oxygen transport, ~15% in myoglobin, and the remainer stored in readily mobilized pools [110]. Copper, likewise, serves as a vital cofactor for electron transfer reactions, most notably in cytochrome C oxidase (or Complex IV), the terminal enzyme in the electron transport chain. Complex IV reduces molecular oxygen to water while driving proton translocation across the inner mitochondrial membrane, thereby generating the electrochemical gradient necessary for adenosine triphosphate (ATP) synthesis [111]. Despite their essential roles, iron, copper, and other transition metals can facilitate the formation of highly reactive hydroxy radicals (•OH) through Fenton (1) and Fenton-like (2) reactions [112]. In these reactions, ferrous iron (Fe^2+^) or cuprous (Cu^+^) donate an electron to H_2_O_2_ yielding ferric iron (Fe^3+^) or cupric (Cu^2+^), hydroxide ion (OH^−^), and a •OH, which is highly reactive and capable of damaging lipids, proteins, and DNA [113,114,115].

Fe^2+^ + H_2_O_2_ → Fe^3+^ + OH^−^ + •OH (1)

Cu^+^ + H_2_O_2_ → Cu^2+^ + OH^−^ + •OH (2)

As part of their antioxidant properties, flavonoids chelate transition metals through hydroxyl and carbonyl groups, forming stable complexes that sequester metals and prevent their participation in redox reactions such as the Fenton reaction, thereby limiting oxidative stress [116]. Quercetin, a flavonol, is particularly well known for its ability to chelate a range of transition metals including Al(III), Co(II), Cu(II), Fe(II), Fe(III), Mo(IV), Pb(II), Tb(III), and Zn(II) [116]. Flavonoids can also neutralize peroxynitrite (ONOO^−^), a reactive nitrogen specie (RNS) generated by the rapid reaction of NO• with superoxide (O_2_•^−^) [117]. This is especially relevant in HTN where Ang II increases superoxide production via NOX, thereby reducing the bioavailability of the potent vasodilator NO [118].

#### 5.2.2. Regulation of Cellular Pathways

Beyond their direct chemical actions, flavonoids exert indirect effects by modulating cellular pathways involved in inflammation, oxidative stress, and redox homeostasis [119]. One of the best-characterized mechanisms is the inhibition of the NF-κB pathway, a central regulator of inflammatory responses [120]. In resting cells, NF-κB remains sequestered in the cytoplasm bound to inhibitory proteins such as IκBα. In the canonical pathway, NF-κB activation is triggered by diverse stimuli including pro-inflammatory cytokines, bacterial products, and physical or chemical stressors [121]. Upon activation, the IκB kinase (IKK) complex phosphorylates IκBα at two N-terminal serine residues, marking it for ubiquitin-dependent degradation. The uninhibited NF-κB complex then rapidly translocates to the nucleus where it binds DNA and initiates the transcription of pro-inflammatory genes.

Flavonoids have been shown to modulate NF-κB signaling through multiple mechanisms that collectively suppress its activation and downstream inflammatory gene expression. Several flavonoids, such as quercetin, luteolin, and epigallocatechin gallate (EGCG), have been shown to inhibit phosphorylation of IκBα by targeting the IKK complex, thereby preventing its degradation [122,123,124]. Additionally, quercetin and luteolin interfere with NF-κB translocation into the nucleus and inhibit it from binding to its DNA response elements [123,125].

In contrast to NF-κB, which amplifies inflammation and oxidative stress, the nuclear factor erythroid 2-related factor (NRF)2 pathway serves as a complementary system that enhances cellular antioxidant and anti-inflammatory defenses [126,127,128]. Under basal conditions, NRF2 is sequestered in the cytoplasm by Kelch-like ECH-associated protein (Keap)1 which targets it for ubiquitination and proteasomal degradation. Upon activation by oxidative or electrophilic stress, NRF2 dissociates from Keap1 and translocates to the nucleus where it binds to antioxidant response elements (AREs) to initiate the transcription of cytoprotective genes. These include heme oxygenase (HO)-1, GPx, SOD, and CAT. Increasing evidence suggests that flavonoids can activate NRF2 signaling thereby shifting the cellular environment from a pro-oxidative to cytoprotective state [129,130]. For example, several flavonoids have been shown to promote NRF2 activation in an extracellular signal-regulated kinases (ERK)1/2-dependent manner [131,132].

Another important mechanism by which flavonoids regulate cellular redox balance is through the suppression of NOX, a major enzymatic source of that contributes to the downstream activation of NF-κB [133]. In addition to promoting inflammation, NF-κB activation also upregulates inducible NO synthase (iNOS), increasing NO production that can react with O_2_•^−^ to form ONOO^−^, thereby amplifying oxidative and nitrosative stress. Flavonoids have been shown to inhibit both NOX and iNOS expression and activity, reducing the production of O_2_•^−^ and NO while limiting ONOO^−^ formation [134].

### 5.3. Flavonoids in the Context of Hypertension and Intestinal Permeability

Mounting evidence suggests that HTN and IP share overlapping oxidative and inflammatory mechanisms that can be modulated by dietary flavonoids. These polyphenolic compounds influence key molecular targets involved in both vascular dysfunction and barrier disruption. In hypertensive states, excessive activation of oxidative pathways contributes to endothelial dysfunction and elevated vascular tone, while in the intestine, similar processes compromise TJ integrity and promotes translocation of pro-inflammatory mediators [135,136,137,138]. Through their combined antioxidant and signaling actions, flavonoids have emerged as promising agents capable of attenuating HTN-associated alterations in IP.

#### 5.3.1. Flavonoids and Hypertension

The antihypertensive effects of flavonoids are largely attributed to their abilities to modulate oxidative and inflammatory pathways that underlie endothelial dysfunction. Activation of RAS by Ang II stimulates NOX isoforms such as NOX1 and NOX2, leading to increased ROS generation and downstream activation of NF-κB-dependent inflammatory signaling. Increased ROS reduces NO bioavailability, thereby inhibiting endothelium-dependent vasodilation, and inflammation contributes to detrimental structural alterations in the vasculature [139,140]. Flavonoids can counteract these effects through both direct antioxidant actions and regulation of redox-signaling pathways. Mechanistically, flavonoids have been shown to decrease NOX activity and enhance the expression and activity of endothelial NOS (eNOS), thereby improving vasodilatory effects [141,142,143,144,145]. Concurrently, flavonoids suppress Ang II-induced NF-κB-mediated transcription of pro-inflammatory cytokines and adhesion molecules which limits inflammation and remodeling [145,146]. Certain subclasses of flavonoids such as anthocyanins have also been shown to inhibit ACE activity and downregulate AT_1_R [147]. All together, these mechanisms converge to restore endothelial homeostasis, reduce vascular tone, and mitigate the oxidative and inflammatory stress brought on by the hypertensive state.

These mechanistic findings have been substantiated in preclinical models of HTN where flavonoid supplementation lowers BP, attenuates vascular oxidative stress, and improves endothelial function [148,149,150,151,152,153,154,155]. In SHRs, intravenous administration of 3-hydroxyphenylacetic acid (3-HPAA), a microbial metabolite derived from several flavonoids, produced rapid and significant reductions in SBP and DBP within five minutes of administration at their lowest dose of 0.1 mg/kg [153]. Similarly, in an Ang II-infusion model, trifolin, a kaempferol glycoside, was shown to attenuate Ang II-induced increases in BP, pulse wave velocity, and aortic collagen deposition, reflecting improved vascular compliance and reduced remodeling [149]. These findings from rodent studies demonstrate that both native flavonoids and their metabolites exert protective effects against HTN consistent with those described in mechanistic studies.

Parallel findings have been reported in human studies investigating flavonoid-rich foods and isolated compounds. Numerous population-based and randomized controlled trials have demonstrated that regular consumption of flavonoid-rich berries, cocoa, and tea can modestly reduce BP [156,157,158,159,160,161]. Meta analyses of these interventions consistently report decreases of approximately 2–4 mmHg in both SBP and DBP [162,163,164]. This is important as decreasing SBP by 5 mmHg has been associated with a 10% lower risk of cardiovascular events [165]. Evidence for specific subclasses, such as flavonols and anthocyanins, remains mixed: several studies report significant reductions in BP while others observe no effects, likely reflecting differences in dose, duration, bioavailability, and participant characteristics [166,167,168]. Nevertheless, when considered collectively, clinical and meta-analytic evidence aligns with preclinical data that support the capacity of flavonoid-rich interventions to improve vascular function and attenuate HTN through antioxidant and anti-inflammatory mechanisms.

#### 5.3.2. Flavonoids and Intestinal Permeability

Under pathological conditions, excessive production of ROS and activation of inflammatory pathways such as NF-κB promote TJ disruption, cytoskeletal contraction, and epithelial cell damage [169,170,171,172]. These alterations increase paracellular permeability, facilitating the translocation of luminal antigens and endotoxins that perpetuate systemic inflammation. Emerging evidence indicates that flavonoids play a crucial role in maintaining intestinal barrier integrity by counteracting oxidative stress and inflammation, two major drivers of increased IP and intestinal barrier dysfunction. For example, Ma et al. [169] demonstrated that curcumin (5 μM) prevented TNF-α-induced (10 ng/mL) reductions in TEER and ZO-1 expression by inhibiting NF-κB activation in Caco-2 cells. These effects were accompanied by downregulation of pro-inflammatory mediators including TLR-4, TLR-2, IL-1β, and TNF-α. The anti-inflammatory and antioxidant roles of flavonoids were further substantiated in a recent study by Feng et al. [173] in which LPS-challenged (1 mg/kg) laying hens fed a diet supplemented with quercetin (0.4 mg/kg) for 12 weeks showed enhanced antioxidant capacity, reduced expression of IL-1β and TLR-4, and increased expression of CLDN-1 and OCLN.

Although human studies examining the effects of flavonoids on IP remain limited, recent findings from the MaPLE (Microbiome mAnipulation through Polyphenols for managing Leakiness in the Elderly) project provide promising evidence. This single-blinded, randomized, crossover, controlled trial in adults over 60 years of age reported significant reductions in serum zonulin concentrations after 8-week polyphenol-rich diet, suggesting improved barrier function [174,175]. Notably, these effects were more pronounced in women than men.

Several in vitro studies have also demonstrated direct effects of individual flavonoids on TJ protein expression. Suzuki et al. [176,177] observed increased expression of ZO-2 and CLDN-4 in Caco-2 cells treated with kaempferol alone, while quercetin alone enhanced CLDN-4 expression and TEER in a dose-dependent manner. In subsequent work, the same group showed that naringenin enhanced protein expression of OCLN, ZO-2, CLDN-1, and CLDN-4, with CLDN-4 regulation partially mediated by the transcription factor Sp1 [178]. Interestingly, naringenin also induced heat shock protein (HSP)70 expression in Caco-2 cells, suggesting a potential protective mechanism through improved protein folding and cellular homeostasis [178,179].

Together, findings from cellular, animal, and emerging human studies indicate that flavonoids preserve intestinal barrier integrity through coordinated antioxidant and anti-inflammatory actions. By stabilizing TJ complexes, suppressing NF-κB-mediated cytokine expression, and enhancing NRF2-dependent antioxidant defenses, flavonoids mitigate epithelial injury and limit the translocation of pro-inflammatory mediators into systemic circulation. These effects parallel the vascular benefits observed in HTN in which flavonoids similarly modulate oxidative and inflammatory signaling to restore endothelial function. The convergence of these mechanisms underscores the potential of flavonoids as cross-system modulators.

#### 5.3.3. Flavonoids: The Cross-System Modulators

Hypertension and IP are increasingly recognized as interconnected pathological processes driven by overlapping oxidative and inflammatory mechanisms. Disruption of the intestinal barrier allows luminal antigens and endotoxins to enter systemic circulation, promoting chronic low-grade inflammation that contributes to endothelial dysfunction and elevated vascular tone. Conversely, HTN-associated vascular inflammation and oxidative stress may further impair intestinal integrity, creating a self-perpetuating cycle of injury. These bi-directional interactions are mediated by shared molecular pathways, including activation of NF-κB, NOX, and iNOS, as well as excessive production of ROS and RNS that diminish NO bioavailability, compromising barrier and vascular function.

Flavonoids act at multiple points within this network to restore redox balance and limit inflammatory signaling. In intestinal epithelium, they stabilize TJ proteins, inhibit NF-κB activation, and activate NRF2-dependent antioxidant responses, thereby reducing permeability and inflammatory mediator leakage. In vascular tissue, flavonoids suppress NOX activity, enhance eNOS function, and reduce iNOS expression, improving NO bioavailability and promoting endothelial relaxation. Through these complementary actions, flavonoids disrupt the positive feedback loop between barrier dysfunction and vascular inflammation, mitigating HTN-associated IP.

**Figure 4:**
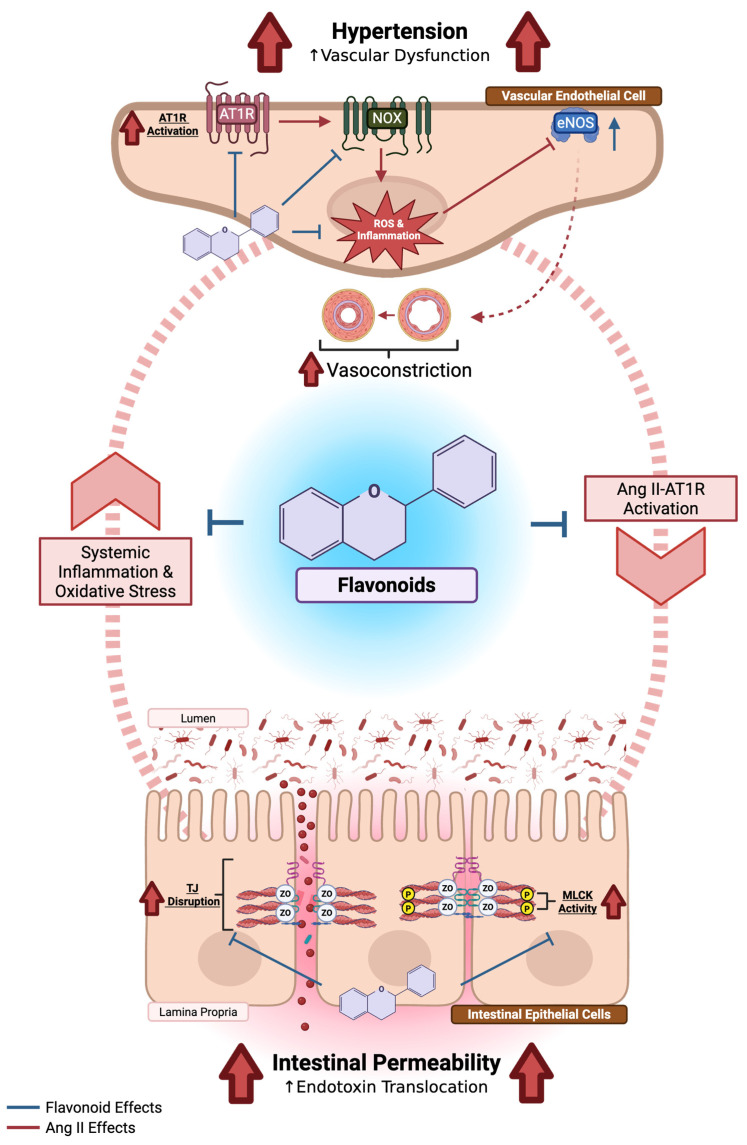
Flavonoids interrupt the pathological feedback loop between hypertension and intestinal permeability. Angiotensin (Ang) II—Ang II Type 1 Receptor (AT_1_R) signaling in vascular and intestinal epithelial cells promotes NADPH oxidase (NOX)-derived reactive oxygen species (ROS) generation and NF-κB-mediated inflammation which collectively reduce endothelial nitric oxide synthase (eNOS) activity and impair vasodilation. Concurrently, this pathway increases myosin light chain kinase (MLCK) activity and disrupts tight junction (TJ) structure by decreasing TJ protein expression and localization. Together, these processes contribute to endothelial dysfunction, vasoconstriction, and increased intestinal permeability. The resulting luminal antigen translocation triggers systemic inflammation and oxidative stress, further exacerbating vascular injury. Flavonoids counteract these effects by inhibiting NOX, NF-κB, and MLCK activity, enhancing eNOS function, and stabilizing TJ integrity, thereby restoring vascular and intestinal homeostasis. Up arrow indicate increase; blunt-ended arrow indicate inhibition. Created with BioRender.com.

Preclinical evidence demonstrates this dual protection clearly. Baicalin, a major flavone derived from *Scutellaria baicalensis Georgi*, lowered BP in SHR while preserving colonic expression of OCLN and ZO-1 and reducing pro-inflammatory mediators including TLR-4, IL-1β, and TNF-α when supplemented at 100 mg/kg/day [180]. These findings align with a recent study by Li et al. [88] in which SHRs treated with unripe apple polyphenol extracts (10–250 mg/kg/day for eight weeks) showed improved BP, enhanced expression of Tjp1 and OCLN, and healthier colonic morphology, accompanied by reductions in TNF-α, TLR-4, and IL-6. Altogether, these studies illustrate how flavonoids function as cross-system modulators, coordinating antioxidant and anti-inflammatory defenses across intestinal and vascular tissues. A conceptual summary of these interactions is illustrated in Figure 4.

## 6. Conclusions and Future Prospects

The evidence reviewed here highlights the intestinal barrier as an overlooked but potentially transformative target in HTN research. The interplay between vascular dysfunction and IP reflects not merely a coincidence of pathology, but a convergence of shared redox and inflammatory mechanisms. Recognizing and addressing this interaction opens new avenues for integrative prevention and therapy beyond BP control alone.

Flavonoids have long been recognized for their cardiovascular benefits and now emerge as cross-system modulators that reinforce both vascular and intestinal integrity. Through antioxidant, anti-inflammatory, signaling-regulatory actions, flavonoids stabilize TJ complexes, preserve endothelial function, and counteract the molecular divers of HTN-associated barrier dysfunction. While the collective preclinical and clinical evidence underscores their potential, significant knowledge gaps remain. Human studies investigating flavonoid-mediated modulation of IP are limited, and factors such as sex, age, and race remain markedly underrepresented. Considering that Ang II-driven HTN manifests differently in females, that antihypertensive drug efficacy varies across racial and ethnic groups, and that intestinal barrier function declines with age, addressing these gaps is essential to improve generalizability and translational relevance.

Future studies should integrate mechanistic, translation, and population-based approaches to clarify the causal relationships between HTN and IP and to determine whether targeting the intestinal barrier can meaningfully modify BP trajectories. Several priorities emerge from the current evidence. First, mechanistic studies are needed to delineate the precise cellular pathways through which Ang II-AT_1_R signaling disrupts TJ structure in vivo, including MLCK activation, cytoskeletal dynamics, and epithelial redox imbalance. Parallel work should determine how specific flavonoid subclasses and their circulating metabolites modulate these pathways, particularly in physiologically relevant concentrations.

Second, limitations in current biomarkers of IP must be addressed. Standardization of zonulin, LPS/LBP, DAO, and D-lactate assays with validation against more rigorous permeability tests will be essential for improving reproducibility and cross-study comparisons. Third, future preclinical studies should move beyond young male rodent models. Sex differences, aging, and genetic background influence both HTN development and mucosal barrier function yet remain understudied. Incorporating female animals, aged cohorts, and models of salt-sensitive or metabolic HTN will better capture clinically relevant variability. Fourth, translational studies must account for the complexity of flavonoid metabolism. Research directly comparing parent compounds to microbial and hepatic metabolites and using physiologically achievable dietary doses will help bridge the gap between experimental findings and real-world feasibility. Finally, well-controlled clinical trials are needed to determine whether flavonoid-rich dietary interventions can improve IP and BP concurrently. Such trials should incorporate longitudinal IP biomarkers, vascular function measures, and stratification by sex, race, age, microbiome composition, and metabolic status to identify individuals most likely to benefit. Collectively, these recommendations will advance the field from associated evidence toward actionable therapeutic strategies and help determine whether improving intestinal barrier integrity can serve as a viable target for HTN prevention and management.

## Figures and Tables

**Table 1:** Summary of studies on blood pressure and intestinal permeability in angiotensin II-infusion model of hypertension.

Animal/Strain	Ang II Dose	Measure of Intestinal Permeability	Other Measures	Reference
8-wk-old-Sprague-Dawley male rats	200 ng/kg/min	**FITC-Dextran 4 kDa:** ↑ **Protein Expression:** Small Intestine: ↓ Tjp1, ↓ Cgn, - CLN, - CLDN4 Proximal Colon: ↓ Tjp1, ↓ Cgn, ↓ OCLN, ↓ CLDN4	**Histology:** Morphology: ↑ Fibrotic Area, ↑ Tunica Muscularis, ↓ Goblet Cells, - Villi Length Inflammatory Cells: ↑ CD3^+^, ↑ CD68^+^, ↑ Iba1^+^	[23]
8-wk-old Sprague-Dawley male rats	200 ng/kg/min (4 weeks)	Not measured	**Histology:** Morphology: ↑ Fibrotic Area, ↑ Tunica Muscularis, ↓ Goblet Cells, - Villi Length	[69]
8-wk-old C57BL/6J male mice	1000 ng/kg/min (4 weeks)	**FITC-Dextran:** ↑ **Gene Expression:** ↓ OCLN, ↓ ZO1, - CLDN4	**FACS:** ↑ Th17	[24]
3-wk-old C57BL/6J male mice	0.25 mg/kg/d (4 weeks)	**Gene Expression:** ↓ Tjp1	**Gene Expression:** ↑ IL17, ↑ IL6, ↑ Tnfα, ↑ Col3a	[70]
10-12-wk-old C3H/HeJ^Lps-d^ and C3H/HeOuJ mice	1000 ng/kg/min (4 weeks)	**FITC-Dextran:** ↑ **Immunolocalization:** ↓ OCLN, ↓ ZO1, ↓ CLDN4	**Gut Bacteria Translocation:** ↑	[71]

Symbols: ↑, upregulation; ↓, downregulation; -, no change. Abbreviations: Ang II, angiotensin II; CLDN, Cgn, cingulin; claudin; OCLN, occludin; FITC, fluorescein isothiocyanate; Tjp1, tight junction protein 1; ZO1, zonula occludens 1. Note: Tjp1 and ZO1 are the same protein; however, we have preserved the names used by the original authors.

**Table 2:** Summary of studies examining intestinal permeability in individuals with hypertension.

Sample Size	Groups *n* (% female)	Age (y)	IP Compared to Reference	Reference
N = 40	HTN: 22 NT: 18	Not Reported	Plasma I-FABP: ↑ Plasma LPS: ↑ Plasma Zonulin: ↑	[24]
N = 357	HTN: 106 (42%) NT: 251 (48%)	HTN: 63 (57–72) NT: 56 (46–63)	Serum DAO: ↑ Serum LPS: ↑ Serum D-Lactate: -	[79]
N = 151	HTN: 27 (78%) Pre-HTN: 61 (32%) NT: 69 (62%)	HTN: 22.2 ± 0.3 Pre-HTN: 22.1 ± 0.2 NT: 21.9 ± 0.3	Plasma zonulin: ↑	[80]

Symbols: ↑, upregulation; -, no change. Abbreviations: DAO, diamine oxidase; HTN, hypertension; I-FABP, intestinal fatty acid binding protein; IP, intestinal permeability; LPS, lipopolysaccharide; NT, normotensive. Age is reported as either median (interquartile range) or mean ± standard deviation based on the original authors.

**Table 3:** Summary of studies using RAS-targeting drugs on intestinal permeability and related outcomes.

Drug	Target	Dose (Duration)	Animal & Disease Model	Measures of Intestinal Permeability & Pathology	Reference
Captopril	ACE Inhibitor	30 mg/kg/day (8 weeks)	Male Spontaneously Hypertensive Rats	**Morphology:** ↓ Muscle Thickness, ↑ Villi Length **Protein Expression:** ↑ TJP1, ↑ OCLN	[88]
Enalaprilat	Ace Inhibitor	14.5 or 145 µg/day (14 days)	Male C57BL/6 mice DSS-induced colitis	**Gene Expression:** ↓ Pro-Collagen (α1), ↓ Pro-Collagen (α2), ↓ TGF-β1 **Protein Expression:** ↓ TGF-β1	[94]
Enalaprilat	ACE Inhibitor	6.25 mg/kg/every 12 h (21 days)	Male IL-10^−/−^ (C57BL/6) mice NSAID- induced colitis	**Transepithelial Resistance:** - **FITC-Dextran 4 kDa:** ↓ **Immunofluorescence Staining:** ↑ OCLN, ↑ ZO1	[89]
Losartan	AT1R Blocker	10 mg/kg/day (2 weeks prior to colitis induction and for the remainder)	Male C57BL/6 J mice TNBS-induced colitis	**FITC-Dextran 4 kDa:** ↓ **Apoptotic Index:** ↓ **Protein Expression:** ↓ BAX, ↑ BCL-2, ↓ Caspase-3	[90]
Captopril	ACE Inhibitor	85 mg/day (4 weeks)	Male Spontaneously Hypertensive Rats	**FITC-Dextran 4 kDa:** ↓ **Morphology:** ↓ Fibrotic Area, ↓ Tunica Muscularis,-Goblet Cells, ↑ Villi Length	[23]
Losartan	AT1R Blocker	20 mg/kg/day (5 weeks)	Male Spontaneously Hypertensive Rats	**Gene Expression:** ↑ OCLN, ↑ ZO1, ↑ MUC-2, - MUC-3 **Plasma:** ↓ LPS	[91]
Losartan	AT1R Blocker	5, 10, or 25 mg/kg (3 injections leading up to measurements)	Male Sprague-Dawley Rats—LPS, repeated WAS, and CRF-induced IBS	**Colonic Permeability:** ↓ in a dose-dependent manner	[92]

*Symbols:* ↑, upregulation; ↓, downregulation; -, no change. *Abbreviations:* ACE, angiotensin converting enzyme; AT1R, angiotensin II type 1 receptor; BAX, BCL-2 associated-x; BCL-2, B-cell leukemia/lymphoma 2; DSS, dextran sulfate sodium; FITC, Fluorescein isothiocyanate; LPS, lipopolysaccharide; MUC-2, mucin 2; MUC-3, mucin 3; OCLN, occludin; TGF-β1, transforming growth factor beta 1; TJP1, tight junction protein 1; TNBS, trinitrobenzene sulfonic acid; ZO1, zonula occludens 1.

## Data Availability

No new data were created or analyzed in this study.

## Abbreviations

The following abbreviations are used in this manuscript:

3-HPAA	3-hydroxyphenylacetic
ACE	Angiotensin Converting Enzyme
Ang II	Angiotensin II
ARBs	Angiotensin II receptor blockers
AREs	Antioxidant response elements
AT1R	Angiotensin II Type 1 Receptor
AT2R	Angiotensin II Type 2 Receptor
BP	Blood Pressure
BPE	Blueberry polyphenol extract
CD	Crohn’s Disease
cGMP	Cyclic guanosine monophosphate
CLDN	Claudin
COX	Cyclooxygenase
CVD	Cardiovascular Disease
DAO	Diamine Oxidase
DASH	Dietary Approach to Stop Hypertension
DBP	Diastolic Blood Pressure
DSS	Dextran sodium sulfate
ECGC	Epigallocatechin gallate
FITC	Fluorescein isothiocyanate
HAT	Hydrogen atom transfer
HSP	Heat Shock Protein
HTN	Hypertension
IECs	Intestinal Epithelial Cells
IFN	Interferon
IKK	IκB kinase
iNOS	Inducible nitric oxide synthase
IP	Intestinal Permeability
JAK/STAT	Janus kinase/signal transducer of activated transcription
JAMs	Junctional Adhesion Molecules
Keap1	Kelch-like ECH-associated protein 1
LPS	Lipopolysaccharide
MaPLE	Microbiome mAnipulation through Polyphenols for managing Leakiness in the Elderly
MDCK	Madine-Darby Canine Kidney
MLC	Myosin Light Chain
MLCK	Myosin Light Chain Kinase
NAC	N-acetylcysteine
NADPH	Nicotinamide adenine dinucleotide phosphate
NF-κB	Nuclear Factor Kappa-Light-Chain-Enhancer of Activated B cells
NOX	NADPH Oxidase
NO	Nitric Oxide
Nrf2	Nuclear factor erythroid 2-related factor 2
OCLN	Occludin
PDTC	Pyrrolidine Dithiocarbamate
PUMA	p53 upregulated modulator of apoptosis
RAS	Renin–Angiotensin System
ROS	Reactive Oxygen Species
SBP	Systolic Blood Pressure
SD	Sprague-Dawley
SET	Single electron transfer
SHRs	Spontaneously Hypertensive Rats
TEER	Trans-Epithelial Electrical Resistance
TGF	Transforming growth factor
TJ	Tight Junction
TJP1	Tight junction protein 1
TLR	Toll-like Receptor
TNBS	2,4,6-trinitrobenzensulphonic acid
TNF	Tumor Necrosis Factor
UC	Ulcerative Colitis
WKY	Wistar Kyoto
XO	Xanthine Oxidase
ZO	Zonula Occludens
